# Partial uncinatectomy combined with anterior cervical discectomy and fusion for the treatment of one-level cervical radiculopathy: analysis of clinical efficacy and sagittal alignment

**DOI:** 10.1186/s12891-021-04680-0

**Published:** 2021-09-12

**Authors:** Haimiti Abudouaini, Tingkui Wu, Hao Liu, Beiyu Wang, Hua Chen, Chengyi Huang, Ying Hong, Yang Meng

**Affiliations:** 1 Department of Orthopedic Surgery, West China Hospital, Sichuan University, No. 37 Guo Xue Xiang Rd, Chengdu, China; 2 Department of Anesthesia and Operation Center, West China school of Nursing, West China Hospital, Sichuan University, Chengdu, China

**Keywords:** Anterior cervical discectomy and fusion, Incinatectomy, Uncovertebral joint, Sagittal alignment, Cervical spondylotic radiculopathy

## Abstract

**Background:**

Biomechanical studies have demonstrated that uncovertebral joint contributes to segment mobility and stability to a certain extent. Simultaneously, osteophytes arising from the uncinate process are a common cause of cervical spondylotic radiculopathy (CSR). For such patients, partial uncinatectomy (UT) may be required. However, the clinical efficacy and sagittal alignment of partial UT during anterior cervical discectomy and fusion (ACDF) have not been fully elucidated.

**Methods:**

A total of 87 patients who had undergone single level ACDF using a zero-profile device from July 2014 to December 2018 were included. Based on whether the foraminal part of the uncovertebral joint was resected or preserved, the patients were divided into the ACDF with UT group (*n* = 37) and the ACDF without UT group (*n* = 50). Perioperative data, radiographic parameters, clinical outcomes, and complications were compared between the two groups.

**Results:**

The mean follow-up was 16.86 ± 5.63 and 18.36 ± 7.51 months in the ACDF with UT group and ACDF without UT group, respectively (*p* > 0.05). The average preoperative VAS arm score was 5.89 ± 1.00 in the ACDF with UT group and 5.18 ± 1.21 in the ACDF without UT group (*p* = 0.038). However, the average VAS arm score was 4.22 ± 0.64, 4.06 ± 1.13 and 1.68 ± 0.71, 1.60 ± 0.70 at 1 week post operation and at final follow up, respectively, (*p* > 0.05). We also found that the C2-7 SVA and St-SVA at the last follow-up and their change (last follow-up value − preoperative value) in the ACDF with UT group were significantly higher than ACDF without UT group (*p* < 0.05). No marked differences in the other cervical sagittal parameters, fusion rate or complications, including dysphagia, ASD, and subsidence, were observed.

**Conclusions:**

Our result indicates that ACDF using a zero-p implant with or without partial UT both provide satisfactory clinical efficacy and acceptable safety. However, additional partial UT may has a negative effect on cervical sagittal alignment.

## Introduction

Cervical spondylotic radiculopathy (CSR) is one of the most common spinal diseases seen in clinical practice. There is an annual incidence of 107.3 per 100,000 for men and 63.5 per 100,000 for women, with a peak incidence in the fourth and fifth decades of life [1, 2]. CSR is defined as neck pain in a radicular pattern in one or both upper extremities related to compression and/or irritation of one or more cervical nerve roots. Anterior cervical discectomy and fusion (ACDF) has become a widely accepted and time-tested surgical intervention for the treatment of CSR patients who are nonresponsive to conservative treatment [3]. Clinical and radiologic results after ACDF appear to be good [4–6]. However, for CSR patients with foraminal stenosis, it is difficult to achieve complete nerve root decompression only by simple discectomy, and such patients often do not have obvious root symptom relief after surgery or have a recurrence after temporary relief [7–9]. Although uncovertebral osteophytes are reported to be the most common cause of nerve root compression in cervical spondylosis [10, 11], the necessity and optimal surgical method of UT in ACDF surgery have been still controversial.

Due to the absence of anterior plates, it is theoretically possible that zero-profile implants worsen the maintenance of cervical sagittal balance compared to traditional cervical plates and cage implants. Moreover, since it was reported that uncovertebral join contributes to spinal motion segment mobility and stability [12, 13], we hypothesized that resecting the uncinate processes during ACDF using a zero-profile implant may cause postoperative sagittal imbalance. However, previous studies related to uncinatectomy during ACDF mainly focus on efficacy, surgical techniques and complications [2, 7, 9, 14–18] and its effect on the cervical sagittal balance was not fully explored.

Thus, considering the paucity of clinical data in this field, a retrospective analysis was performed to investigate the effect of uncinectomy on sagittal parameters by comparing the clinical and radiologic outcomes after ACDF with partial uncinatectomy (UT) versus ACDF without UT.

## Materials and methods

### Patient recruitment and inclusion criteria

We retrospectively reviewed all one-level ACDF cases with a zero-profile device (Zero-P, Synthes GmbH, Switzerland) for spondylotic radiculopathy from July 2014 to December 2018 performed by the same senior spine surgeon in our department with a minimum of 1-year clinical follow-up. Indications for surgery included patients with radiculopathy secondary to herniated disc, spondylosis, or a combination of both that was refractory to conservative treatment.

The inclusion criteria included the following: (1) patients with symptoms of degenerative cervical disease; (2) patients who received only single level ACDF; and (3) a follow-up period greater than 12 months. The exclusion criteria were as follows: (1) patients who had a history of previous cervical spine surgery, fractures, tumours, etc.; (2) patients who underwent multilevel ACDF; (3) patients with myelopathy, congenital cervical malformation, ankylosing spondylitis, severe osteoporosis (T-score ≤ − 2.5), rheumatoid arthritis, cervical infection, pregnancy, metal allergy, or a neuromuscular disease; and (4) Patients with bilateral uncinatectomy; (5) a follow-up period of less than 12 months.

**Fig. 1:**
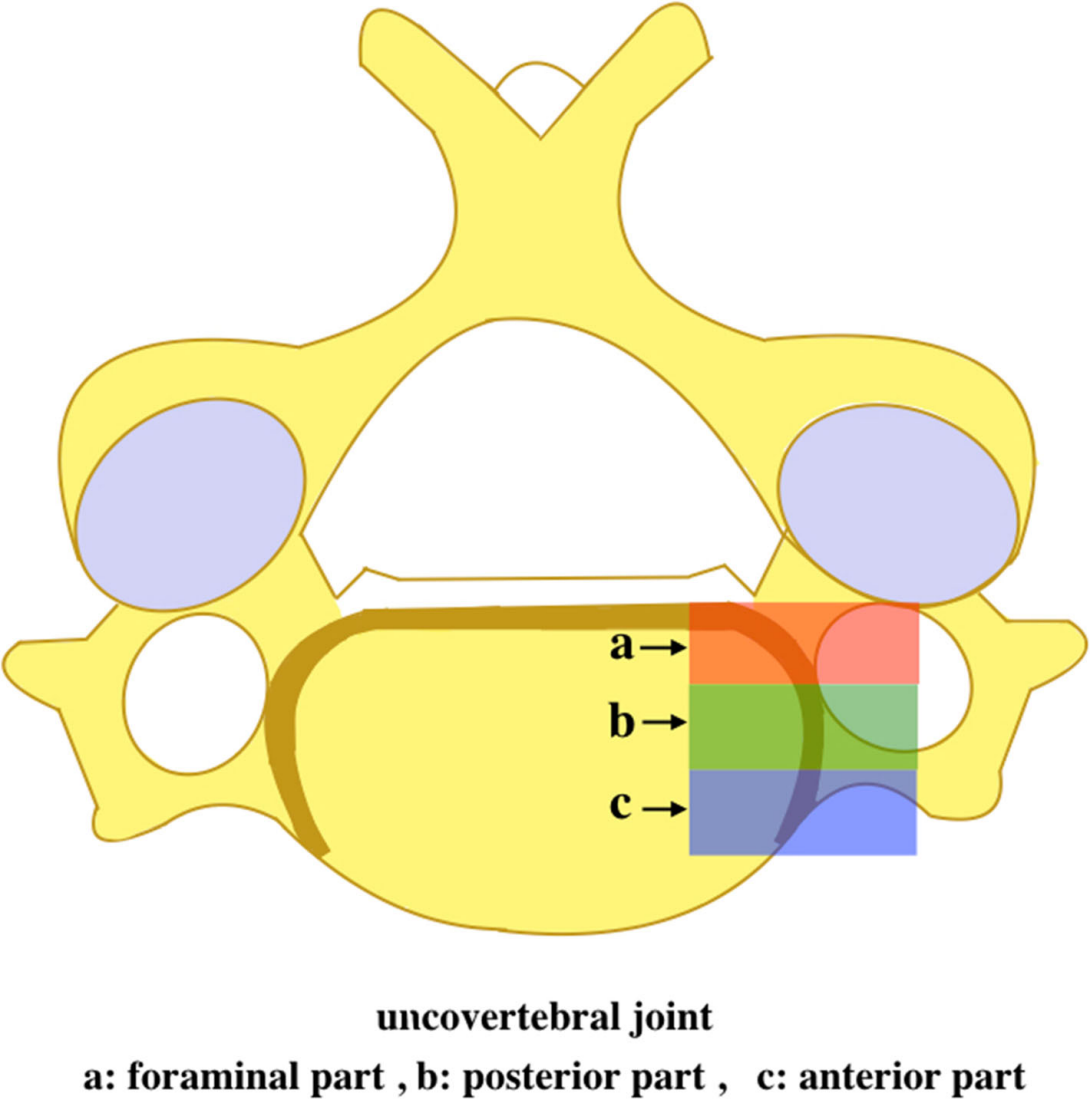
Anatomic schematic drawing of the uncovertebral joint

Partial uncinatectomy was defined as using a high-speed matchstick burr to remove the foraminal part of the uncovertebral joint [19], while no uncinatectomy was defined as preserve the uncinate process intact (Fig. 1). This was confirmed with postoperative computed tomography scans and medical records.

### Surgical technique

A standard Smith-Robinson anteromedial left-sided cervical approach was used in all cases. After sufficient exposure, complete discectomy was performed at the index levels by removing the disc tissue, posterior longitudinal ligament and osteophytes to achieve thorough decompression. Evidence of foraminal stenosis due to uncovertebral joint hypertrophy was confirmed by preoperative imaging and intraoperative exploration. For these patients, a high-speed matchstick burr was used to remove the hyperplastic osteophytes and foraminal part of the uncovertebral joint [19]. If the patient had unilateral symptoms and if the radiologic results were consistent, we unilaterally removed the hyperplastic osteophytes and foraminal part of the uncovertebral joint. We used a zero-p implant (Zero-P, Synthes GmbH, Switzerland) filled with a composite synthetic bone graft ( Paoli, CA, USA) implanted into the index levels. The final imaging of the device was performed before the locking head screws were screwed. After the surgery, the incision was sutured layer by layer.

### Clinical outcome assessment

Demographic data, including age, sex, body mass index (BMI), intraoperative blood loss and hospital stay days, were obtained from the patient medical records. Clinical outcomes were assessed by the visual analogue scale (VAS), Neck Disability Index (NDI), and Japanese Orthopaedic Association (JOA) score. All clinical evaluations were collected preoperatively, immediately after surgery, and at the last follow-up.

### Radiological evaluation

**Fig. 2:**
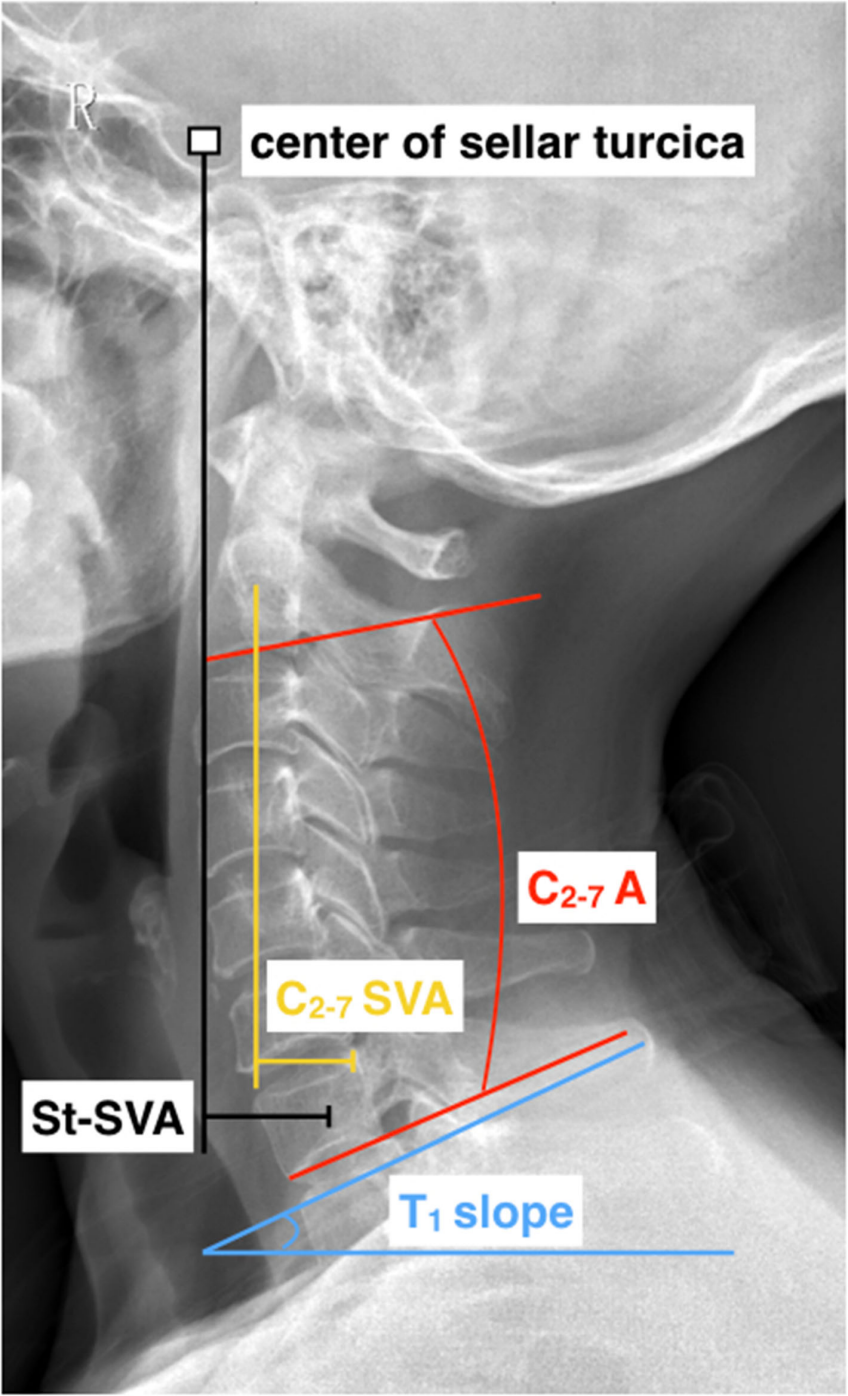
Lateral cervical spine radiograph with an illustration of key cervical sagittal alignment measurements. SVA indicates the sagittal vertical axis. C_2 − 7_ A represents the C2 - C7 angle

Radiological analysis was conducted via lateral radiographs for flexion, extension, and neutral positions. All radiographic measurements were performed by two spine surgeons who did not participate in these surgeries. Cervical lordosis (CL), functional spinal unit angle (FSUA), sagittal vertical axis (C2-7 SVA), centre of the sella turcica–C7 sagittal vertical axis (St-SVA), and T1 slope were recorded according to methods described in previous studies [20, 21] (Fig. 2). CL was measured between the inferior margin of the C2 vertebrae, and the inferior margin of the C7 vertebrae was measured as the C2–7 angle. The FSUA was calculated using the Cobb angle of the adjacent vertebrae to the involved intervertebral disc. The C2–C7 SVA was decided as the length from the posterosuperior corner of C7 and the vertical line from the centre of the C2 body. The centre of the St-SVA was defined as the distance between a plumb line from the centre of the sellar turcica and the centre of the C7 body. The T1 slope was defined as the angle between the T1 superior endplate and a horizontal line.

### Statistical analysis

All statistical analyses were performed using SPSS 25.0 software (SPSS Inc., Chicago, IL, USA). Continuous data are presented as the means ± standard deviation, and the counting data are expressed as (%). The independent t-test and chi-square analysis were used when the normality assumption was satisfied, and the Mann-Whitney U-test was used when the normality assumption was not satisfied in the Kolmogorov-Smirnov test. A *P* value < 0.05 was considered statistically significant.

## Results

### Patient demographics

A total of 87 patients met the inclusion criteria, and 37 patients were included in ACDF with UT group and 50 patients were included in each group. The mean follow-up was 16.86 ± 5.63 and 18.36 ± 7.51 months in the ACDF with UT group and ACDF without UT group, respectively (*p* > 0.05) (Table 1). No significant differences were found in age, sex, BMI, blood loss, hospital stay or level distribution between the two groups (Table 1).

**Table 1:** Demographic and baseline data (mean ± SD)

Groups	ACDF with UT (*N* = 37)	ACDF without UT (*N* = 50)	*p*
Age (years)	51.46 ± 9.47	53.47 ± 10.36	0.206
Gender (female/male)	17/20	22/28	
BMI (kg/m^2^)	23.55 ± 2.64	23.95 ± 2.60	0.478
Smoking (yes/no)	14/23	18/32	
Alcohol (yes/no)	8/29	13/37	
Blood loss (ml)	79.03 ± 51.21	76.62 ± 62.57	0.922
Hospital stay (days)	8.64 ± 2.27	8.24 ± 2.36	0.482
Operation level			
C3/4	2	8	
C4/5	5	8	
C5/6	26	32	
C6/7	4	2	
Follow-up	16.86 ± 5.63	18.36 ± 7.51	0.312

*ACDF *anterior cervical discectomy and fusion, *UT* uncinatectomy, *BMI* body mass index

*p* < 0.05 was the criterion for statistical significance

### Comparison of clinical parameters

There were no significant differences in preoperative JOA, Neck-VAS, or NDI between the two groups. The average preoperative VAS arm score was 5.70 ± 1.05 in the uncinatectomy group and 5.18 ± 1.21 in the non-uncinatectomy group. The preoperative Arm-VAS score in the uncinatectomy group was significantly higher than that in the non-uncinatectomy group (*p* = 0.038, Table 2). All patients showed pain relief and neurologic improvement after surgery, and no significant postoperative differences were found in clinical parameters between the two groups.

**Table 2:** Clinical outcomes of two groups

	ACDF with UT	ACDF without UT	*P*
JOA scores			
preoperative	11.11 ± 2.23	10.90 ± 1.98	0.648
1 week	12.72 ± 1.65	12.44 ± 1.86	0.495
Last follow-up	15.59 ± 1.57	15.72 ± 1.39	0.690
VAS neck score			
preoperative	5.89 ± 1.00	5.66 ± 1.08	0.443
1 week	4.23 ± 0.81	4.16 ± 0.65	0.722
Last follow-up	1.97 ± 1.10	1.64 ± 0.83	0.105
VAS arm score			
preoperative	5.70 ± 1.05	5.18 ± 1.21	0.038*
1 week	4.22 ± 0.64	4.06 ± 1.13	0.451
Last follow-up	1.68 ± 0.71	1.60 ± 0.70	0.621
NDI scores			
preoperative	24.11 ± 3.76	22.96 ± 5.65	0.586
1 week	17.81 ± 3.58	16.76 ± 5.23	0.621
Last follow-up	10.99 ± 4.95	9.71 ± 2.82	0.586

^*^Indicates statistically significant differences (*p* < 0.05)

### Comparison of cervical sagittal alignment

**Fig. 3:**
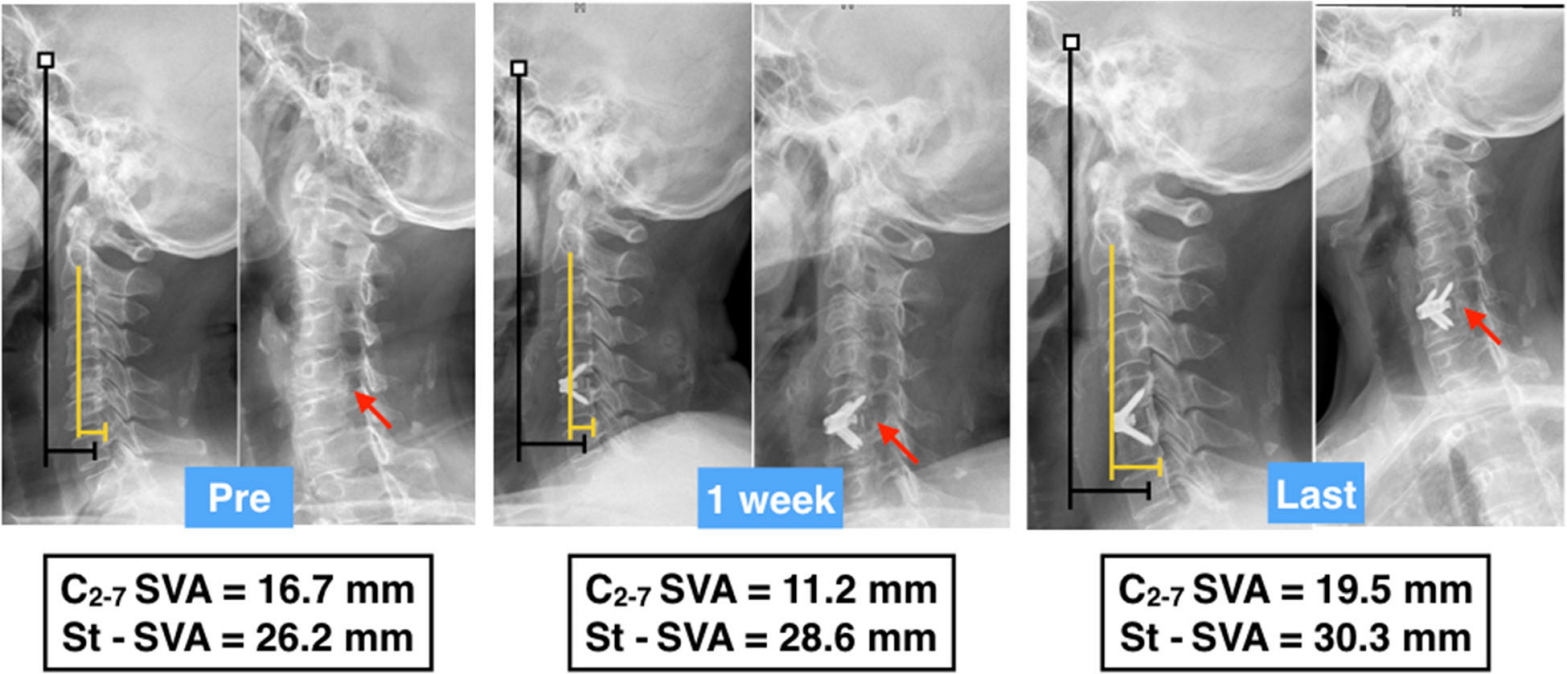
A 58-year-old woman was diagnosed with cervical radiculopathy combined with foraminal stenosis caused by uncovertebral joint hyperplastic osteophytes, which can be seen on preoperative right oblique imaging (red arrow). The patient underwent ACDF with partial uncinatectomy. The improvement of C2-7 SVA and St-SVA was not obvious after the surgery

**Fig. 4:**
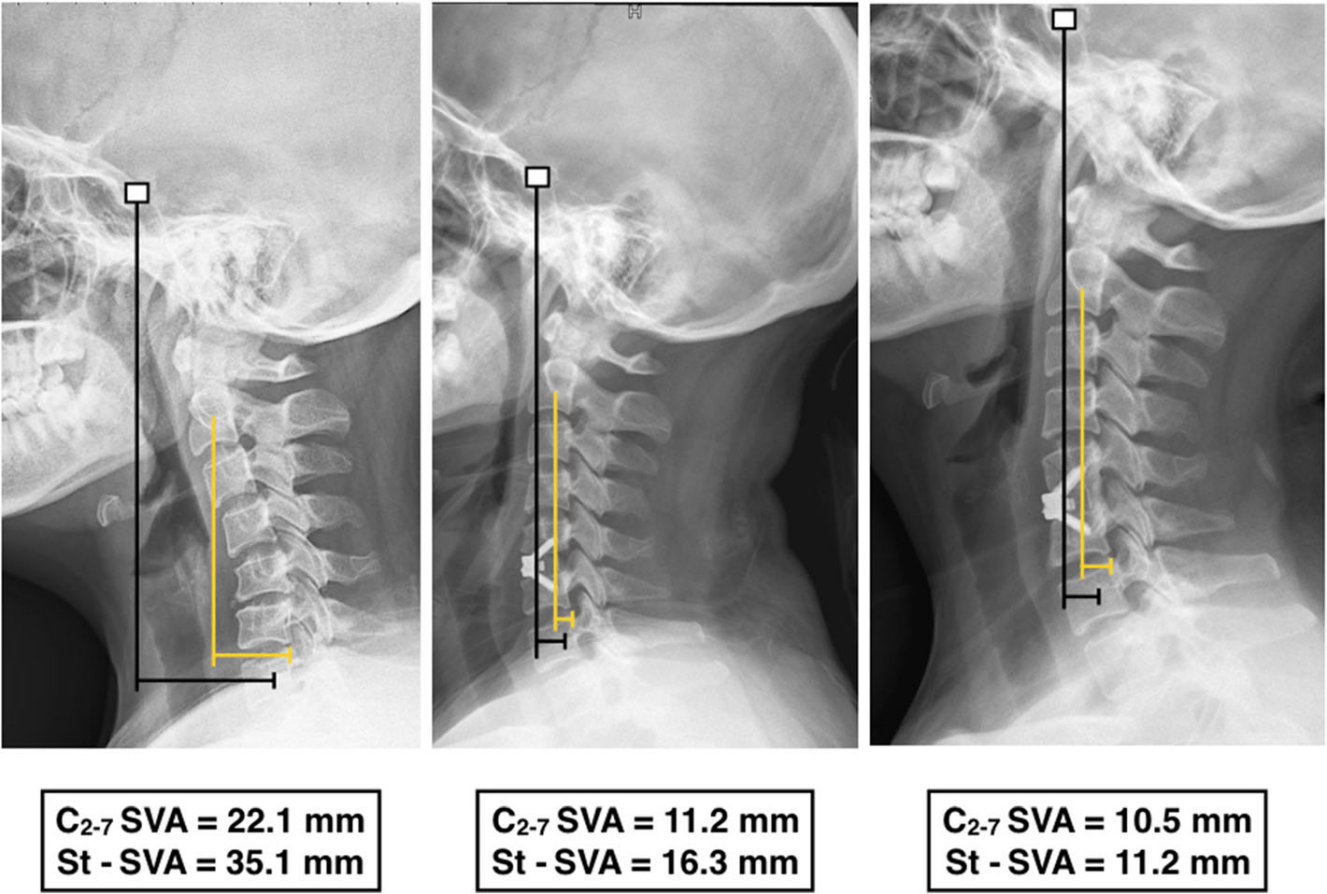
A case from the ACDF without uncinatectomy group. The C2-7 SVA and St-SVA decreased significantly over time

Cervical sagittal alignment parameters are shown in Table 3. With the exception of C2-7 SVA and St-SVA, the other sagittal alignment parameters were similar at various time points (*p* > 0.05). In the uncinatectomy group, the C2-7 SVA was maintained from 18.67 ± 6.08 mm before surgery to 18.62 ± 6.33 mm at the last follow-up, with a mean change value of -0.05 ± 6.22 mm. In the non-uncinatectomy group, it decreased from 19.84 ± 7.00 mm before surgery to 15.75 ± 6.02 mm at the last follow-up, and the mean change value was − 4.09 ± 9.21. There were significant differences between the groups in the C2-7 SVA at the last follow-up (*p* = 0.034) and the mean changes in C2-7 SVA values (*p* = 0.023) (Table 3). In addition, the St-SVA decreased from 28.09 ± 5.83 mm to 26.25 ± 10.64 mm in the uncinatectomy group, with a mean change of -2.49 ± 12.51, and 29.86 ± 6.69 mm to 22.15 ± 8.44 mm in the non-uncinatectomy group at the last follow-up, with a mean change of -7.70 ± 8.44. There were significant differences between the groups in the St-SVA at the last follow-up (*p* = 0.033) and changes in the St-SVA values (*p* = 0.019) (Table 3; Figs. 3 and 4).

**Table 3:** Radiographic assessments of patients in three groups (mean ± SD)

Group	ACDF with UT	ACDF without UT	*p*
C2-7 A (°)			
preoperative	14.23 ± 5.06	12.93 ± 7.00	0.345
1 week	13.26 ± 8.13	11.96 ± 6.46	0.176
Last follow-up	12.78 ± 7.49	11.25 ± 6.62	0.317
dC2-7 A (°)	-1.45 ± 8.45	-1.69 ± 7.72	0.874
FSUA (°)			
preoperative	3.24 ± 1.93	3.37 ± 1.89	0.875
1 week	3.12 ± 1.88	3.21 ± 1.87	0.826
Last follow-up	3.05 ± 1.80	3.09 ± 1.93	0.914
dFSUA (°)	-0.25 ± 0.58	-0.28 ± 0.59	0.862
C2-7 SVA (mm)			
preoperative	18.67 ± 6.08	19.84 ± 7.00	0.417
1 week	18.32 ± 6.03	18.17 ± 6.99	0.915
Last follow-up	18.62 ± 6.33	15.75 ± 6.02	0.034 *
dC2-7 SVA (mm)	-0.05 ± 6.22	-4.09 ± 9.21	0.023 *
St-SVA (mm)			
preoperative	28.09 ± 5.83	29.86 ± 6.69	0.417
1 week	24.80 ± 6.36	25.17 ± 5.39	0.052
Last follow-up	26.25 ± 10.64	22.15 ± 8.44	0.033 *
dSt-SVA (mm)	-2.49 ± 12.51	-7.70 ± 8.44	0.019 *
T1 slope (°)			
preoperative	27.92 ± 6.66	26.29 ± 6.30	0.229
1 week	26.55 ± 5.19	27.00 ± 5.71	0.709
Last follow-up	26.79 ± 6.20	27.19 ± 7.07	0.784
dT1 slope(°)	-1.18 ± 5.69	0.91 ± 7.23	0.149

*SD* standard deviation, *C2-7 A* C2-7 angle; FSUA, functional spinal unit angle,*C2-7 SVA* C2-7 sagittal vertical axis, *St-SVA* sellar turcica–sagittal vertical axis

d: the difference of parameters at last follow-up and preoperative

*Indicates statistically significant differences (*p* < 0.05)

### Comparison of the complications and fusion rate

During the follow-up period, there were no serious complications that required revision surgery. There were no significant differences in the occurrence of dysphagia, adjacent segment degeneration (ASD) or cage subsidence. At the time of the last follow-up, the fusion rates were 97.30 and 96 % in the two groups, respectively (*p* = 0.743, Table 4).

**Table 4:** Comparison of complications among three groups

Groups	ACDF with UT (*N* = 37)	ACDF without UT (*N* = 50)	*p*
Dysphagia	8.11 % (3)	8.00 % (4)	0.985
ASD	10.81 % (4)	12 % (6)	0.863
Cage subsidence	16.22 % (6)	10 % (5)	0.388
Fusion rates	97.30 % (36)	96 % 48)	0.743

*ACDF *anterior cervical discectomy and fusion, *UT* uncinatectomy

## Discussion

Uncovertebral joint is common site for osteoarthritic changes [22]. These changes manifest as pitting and eburnation of the articular surfaces and distortion of the uncinate process as osteophytic spurring develops. Osteophytes that arise from the posterior aspect of the uncinate process project into the intervertebral foramen and compress its contents. Despite this, there are various opinions on the treatment of CSR patients with foraminal stenosis. Riley et al. thought that with the establishment of spinal stability and the release of abnormal stress stimuli, osteophytes can spontaneously be absorbed [23]. Shen et al. [13] conducted a retrospective study to analyse the necessity of direct uncovertebral joint decompression during ACDF, and they found that ACDF with or without direct uncovertebral joint decompression can provide good clinical results for neck pain with cervical radiculopathy. Cloward considered that all factors that compress nerve roots should be removed in anterior cervical surgery, including uncovertebral osteophytes [24]. Therefore, routine direct uncovertebral joint decompression should not be undertaken during ACDF. In contrast, Park et al. [25] reported that ACDF with uncinatectomy can provide a better long-term outcome with minimal ASD. In our study, there were no significant differences in postoperative VAS arm and neck scores, which indicates that ACDF with uncinatectomy was not superior than ACDF without uncinatectomy in terms of clinical efficacy.

The cervical spine is the part of the spine with the most mobility in the sagittal plane [2]. During the last decade, the study of cervical sagittal balance became highlighted as it links functionality and the surgical outcome [26]. To the best of our knowledge, only one article has reported the effect of uncinatectomy on sagittal balance after one-level ACDF with a cage-and-plate construct [21]. They found that cervical sagittal alignment after ACDF with uncinatectomy was not significantly different from that achieved with ACDF without uncinatectomy; however, the occurrence of subsidence was higher in the ACDF with uncinatectomy group. In this study, similar results in sagittal alignment were obtained. Although a higher cage subsidence rate (8 %) was observed in the ACDF with uncinatectomy group, the difference between the two groups did not reach statistical significance (*p* = 0.388, Table 4). It was reported that various factors including cage type [27] and location [28], distractive force [29], end-plate preparation [30], and particularly, a small degree of segmental instability (micro-motion) [31, 32] may affect subsidence. Lee et al. [33] investigated the effect of UT on the subsidence following ACDF using a anterior plate fixation. The UT, especially uncinate process resection area > 38 %, significantly increase the micro-motion between plate and bone interface and the possibility of subsidence will increased.

C2-7 SVA is thought to be the best indicator of cervical malalignment, and it was reported that it has a close relationship with clinical outcomes after cervical surgery [25,41,42]. As another representative factor for estimating radiological outcome, St-SVA was also demonstrated to be related to postoperative neck pain and health-related quality of life (HRQOL) [28,43,44]. However, C2-7 SVA and St-SVA were not reflected in postoperative neck pain or quality of life in our study. A possible reason for this dissimilarity is that the incidence, severity and mechanisms underlying postoperative neck pain differed between the anterior and posterior cervical surgeries. In our study, C2-7 SVA and St-SVA of ACDF without UT group were restored from 19.84 ± 7.00mm to 15.75 ± 6.02mm and 29.86 ± z6.69mm from 22.15 ± 8.44mm at the last follow-up and this trend was similar with the previous studies [21, 34, 35]. SVA is an important evaluation index of the degree of the overall cervical displacement. Our findings demonstrated that the ACDF itself, which using a zero profile device, can restore the displacement of the center of gravity and sagittal alignment of cervical spine to some extent, which may subsequently reduce the energy expenditure of the neck and shoulder muscles. Besides, ACDF without UT group is superior to ACDF with UT group in terms of improving C2-7 SVA and St-SVA. Although we are unable to analyze exact mechanism in a retrospective study, we speculated a possible reason according to our result: foraminal part of the uncovertebral joint may plays important role in restricting the lateral displacement of lower cervical spine. Further biomechanical studies are needed to verify this.

Clausen et al. [12] analysed the biomechanical significance of uncinate processes and found that if uncinate processes are resected during surgery, primary motion will increase relative to intact motion. Thus, they suggested the use of a fusion procedure to reduce lateral bending instability resulting from resection of the uncinate processes during ACDF. It was reported that the uncovertebral joint contributes 48–60 % of the total stability at each level, with the posterior aspect of the uncovertebral joint providing the greatest stability [13]. Although total uncinatectomy has been reported to be an effective method [15–18], we agree that in the process of uncovertebral joint decompression, removing the hyperplastic osteophytes and foraminal part of the uncovertebral joint would be sufficient to relieve nerve root compression, and the procedure also reduces injury to the vertebral artery to a certain extent. In addition, because the uncovertebral joint has rich venous vessels, serious haemorrhage may be more likely to occur during total uncinatectomy.

### Limitations

Our study has some limitations. First, this is a single-centre, retrospective, nonrandomized controlled study with a relatively small number of cases. Second, the average follow-up of these patients was 17.72 ± 6.78 months, which severely limited our analysis of long-term outcomes. In addition, although two spine surgeons measured the radiological parameters with reference to previously published reports, we acknowledge that potentially inherent radiographic imaging error may be another limitation. Furthermore, it is challenging to precisely evaluate the degeneration of adjacent segments without postoperative MRI imaging of the cervical spine. However, we can still adequately assess ASD according to the abovementioned radiographic criteria. Another major limitation is that we did not include the patients with bilateral uncinatectomy. The main reason is that the number of patients who underwent bilateral uncinatectomy was much smaller than that of patients who underwent unilateral uncinatectomy in our department. Last, we did not analyse the effect of uncinatectomy on the thoracolumbar region or spine-pelvic sagittal balance. We hope future studies, especially biomechanical studies, can answer these questions.

## Conclusions

Our result indicates that ACDF using a zero-p implant with or without partial UT both provide satisfactory clinical efficacy and acceptable safety. However, additional partial UT may has a negative effect on cervical sagittal alignment.

## Data Availability

The datasets used and/or analysed during the current study are available from the corresponding author on reasonable request.

## Abbreviations

CSR: Cervical spondylotic radiculopathy
UT: Uncinatectomy
ACDF: Anterior cervical discectomy and fusion
PCC: Plate-cage construct
Zero-P: Zero-profile device
BMI: Body mass index
VAS: Visual analogue scale
NDI: Neck Disability Index
JOA: Japanese Orthopaedic Association
CL: Cervical lordosis
FSUA: Functional spinal unit angle
SVA: Sagittal vertical axis
St-SVA: Sella turcica–C7 sagittal vertical axis
ASD: Adjacent segment degeneration
HRQOL: Health-related quality of life
